# Research on Damage Detection of Dual-Rotor Synchronous Excitation Mine Screen Beams Based on Strain Mode Difference Vibration Mode Analysis

**DOI:** 10.3390/s24227133

**Published:** 2024-11-06

**Authors:** Xiaohao Li, Yahui Wang, Yang Zhou

**Affiliations:** 1 School of Mechanical Engineering and Automation, Northeastern University, Shenyang 110819, China; 2 Key Laboratory of Vibration and Control of Aero-Propulsion System, Ministry of Education, Northeastern University, Shenyang 110819, China

**Keywords:** strain mode difference mode analysis, damage detection, dual-rotor synchronous excitation, mine screen frame beam, acceleration sensor

## Abstract

The frame beam structure of the mine screen, subjected to various excitations, is a critical component of mining machinery. Its stress is intricate, the operational environment is severe, and damage can lead to catastrophic failures resulting in machinery destruction and fatalities. Based on the characterization of the vibration response of mine screen frame beams with varying degrees of damage at the same location and with the same degree of damage but at different locations, this paper develops a method of strain modal difference vibration pattern analysis and damage feature extraction for the detection of structural damage in beams. This method is based on the sensitivity of the sudden change in vibration strain modal difference to small deformations. This method solves the problem of using the conventional structural finite element analysis or experimental modal analysis methods to obtain the displacement mode, intrinsic frequency, and other characteristics, which make it difficult to effectively identify the actual engineering, with the damage conditions of the damage state and damage location of the mine screen frame beam problems. The feasibility and validity of the engineering application of the concept are demonstrated through instances.

## 1. Introduction

The frame beam structure [1], subjected to various excitations, is a critical component of mining machinery, characterized by intricate stress patterns and a severe operational environment, frequently leading to catastrophic failures and fatalities following damage. To guarantee the long-term safe operation of the frame beam structure under multi-excitation impact conditions, it is crucial to monitor the structural unit and accurately determine the damage location. Currently, the investigation and implementation of technology in this domain have emerged as a prominent study topic both nationally and internationally.

Presently, numerous techniques exist for identifying structural damage, with rapidly advancing approaches including eddy current detection, radiographic detection, magnetic particle detection, and ultrasonic detection, among others [2,3]. Nonetheless, the excitation from multiple sources acting on the mine screen frame beam, coupled with the presence of multi-frequency interference noise, dust, and strong perturbation of loads to detection media such as magnetic levitation and rays, poses significant challenges. Consequently, in practical engineering applications, it is frequently necessary to manually ascertain the approximate location of the damaged unit before conducting a more precise identification. The application of huge, long structural frame beam units is somewhat constrained, rendering the technology predominantly useful for localized damage detection [4].

In the realm of damage identification for beam structures via vibration response analysis, literature [5] proposes the principle that the intrinsic frequency of pipeline structures alters due to damage. It employs finite element simulation to ascertain the intrinsic frequency of pipeline structures and assesses the damage condition of structural members through variations in the intrinsic frequency. Literature [6,7,8] examined the sensitivity of modal vibration alterations to structural damage and conducted damage identification research on cantilever beam structures by vibration modal test analysis. Guo et al. [9] examined the time-domain and time-frequency-domain signals of drilling pumps using deep neural networks, introducing a novel parallel deep neural network approach for drilling pump fault diagnosis, which yielded promising outcomes in the research and application of fault diagnosis for energy equipment. Chen et al. [10] used convolutional neural networks (CNNs) for mechanical system fault diagnosis, leveraging their robust feature extraction and classification skills to accomplish three representative mechanical troubleshooting cases. Bhowmik et al. [11] evaluated the sensitivity of structural modal vibration patterns and frequency response function metrics for detecting structural damage, concluding that frequency response function metrics are more effective in precisely identifying damage through wavelet analysis. Ref. [12] proposed a method that combines intrinsic frequency analysis with ultrasonic detection to ascertain the size and location of structural damage. This approach offers certain advantages in detection efficiency. However, due to the fact that the intrinsic frequency variation is not sensitive to the initial damage of long structures and the error of the determined damage location is large, this method is not widely used in practical engineering. Consequently, it has not been widely applied in practical engineering contexts. Yan et al. [13] and Morteza et al. [14] developed a dynamic model of beam structures subjected to impact loads, investigated the effects of vibration modal properties and elastic deformation on structural damage, and assessed the vibration response mechanisms in the context of structural damage. Esu et al. [15] extracted potential failure time-domain information from mechanical equipment using multiple sensors, achieving fault diagnosis through multi-sensor fusion technology and information processing algorithms, demonstrating exceptional resistance to noise interference. Ref. [16] employed the phenomenon of modal strain energy variation in the damaged unit to investigate the method for identifying damage locations based on the rate of change of modal strain energy. However, its effectiveness in industrial applications is suboptimal, primarily because the displacement modal strain energy lacks sensitivity to minor damages, leading to potential misjudgments.

This paper addresses the limitations of current structural damage detection technologies and the complexities associated with multi-point excitation response issues in long structures. It develops a modal strain energy rate of change damage index and a damage identification algorithm for frame beam structural units, utilizing vibration response analysis of the beam structural unit and the differential rate analysis of change in displacement modes in the tested unit, combined with the strain modal discrepancy constant mutation effect observed during synchronous excitation of the mine sieve frame beam in a damaged state. By formulating a dynamic model where the intrinsic frequency, modal vibration pattern, and displacement response of the mine screen’s frame beam structure serve as dependent variables, while mass, stiffness, and internal damping function as independent variables, this study analyzes the modal alterations in the system’s vibration response induced by damage to the mine screen beam structure under synchronous excitation. It extracts the patterns of change in the physical parameters of the frame beam structure and utilizes the property that minor variations in the strain modal difference during differential computation are amplified. This research is based on the mode shape analysis of strain mode difference for identifying structural damage in the mine screen frame beam, effectively enabling the recognition of the damage degree and the determination of the damage location of the frame beam of the mine screen.

## 2. Damage Identification Algorithm of Beam Element Based on Strain Modal Difference Mode Shape Analysis

According to Ref. [17], when damage occurs in a certain part of the beam structure, stress concentration will occur around the damaged part, and the stress will change sharply with the change in damage degree, but other undamaged parts have no change or little change, and the relative damage position can be approximately ignored. It was known from Ref. [18] that strain is the first derivative of displacement. For beam structure, every displacement mode has corresponding inherent strain distribution state, and strain mode is the inherent characteristic of beam structure [19], which is not affected by external load. Therefore, according to the strain modal principle, the structural damage identification can be carried out on the basis of calculating the strain modal difference of the beam structure.

The modal shape of the beam structure element is the natural equilibrium state of deformation energy when the beam structure vibrates without damping. The equilibrium state does not depend on other equilibrium states; that is, the inherent modes of the frame beam are complementary and coupled, so that the actual displacement response of the beam element is the superposition of various modes. Therefore, the displacement response of the beam element is expressed as the sum of various modes:

(1) $$\left\{X\right\}=\sum_{r=1}^{n}\left\{\tau_{i,r}\left(t\right)\right\}\left\{\phi_{i,r}\left(x\right)\right\},$$

where $\left\{X\right\}$ represents the displacement response vector of the beam structure, $\left\{\tau_{i,r}\right\}$ represents the time-dependent displacement modal coordinates of the r-th order of beam element i, and $\left\{\phi_{i,r}\right\}$ represents the displacement mode of the r-th order of beam element i.

According to the theory of structural dynamics [20], the differential equation for the bending vibration of beam structures can be expressed as

(2) $$\frac{d^{2}}{dx^{2}}\left[EI\frac{d^{2}}{dx^{2}}\left(\sum_{r=1}^{n}\tau_{i,r}\left(t\right)\phi_{i,r}\left(x\right)\right)\right]+\rho \left(x\right)\frac{d^{2}}{dx^{2}}\left(\sum_{r=1}^{n}\tau_{i,r}\left(t\right)\phi_{i,r}\left(x\right)\right)+c\left(x\right)\frac{d}{dx}\left(\sum_{r=1}^{n}\tau_{i,r}\left(t\right)\phi_{i,r}\left(x\right)\right)=f\left(x,t\right)$$

where E is the elastic modulus of the beam structure, I is the cross-sectional moment of inertia of the beam structure, $\rho \left(x\right)$ is the density of the beam structure, and $c\left(x\right)$ is the internal damping of the beam structure.

By taking Equation (1) into Equation (2) and based on the orthogonality of modal shapes, Equation (3) can be obtained as

(3) $$m_{i}{\ddot{\tau }}_{i,r}+c_{i}{\dot{\tau }}_{i,r}+k_{i}\tau_{i,r}=\int_{0}^{L}f_{i}\left(x,t\right)\phi_{i,r}\left(x\right)dx\;\;\;\;r=1,2,\cdots ,n,$$

where $m_{i}=\int_{0}^{L}\phi_{i,r}^{2}\left(x\right)\rho \left(x\right)dx$ is the mass of beam element i, $c_{i}\left(x\right)=\int_{0}^{L}\phi_{i,r}^{2}\left(x\right)c\left(x\right)dx$ is the internal damping of beam element i, $k_{i}=\int_{0}^{L}{\left[\frac{d^{2}}{dx^{2}}\phi_{i,r}\left(x\right)\right]}^{2}EIdx$ is the stiffness of beam element i, and $f_{i}\left(x,t\right)$ is the force acting on beam element i.

For Equation (3), the beam structure is subjected to excitation $f_{i}\left(x,t\right)=F_{i}\left(x\right)e^{j\omega t}$ and solved by the multi-degree of freedom modal analysis theory [21]; the structural displacement response of the beam element can be obtained as

(4) X=x1x2x3⋮xn=∑r=1nτi,rTτi,r−ωr2mi+jωrci+KiejωrtF1F2F3⋮Fn.

If the cross-sectional height of the beam to be tested is H, then based on the geometric relationship of structural mechanics deformation [22], the strain response mode of the beam element can be obtained as

(5) $$\sigma_{x}=\frac{\partial^{2}}{\partial x^{2}}\left[\sum_{r=1}^{n}\tau_{i,r}\left(t\right)\phi_{i,r}\left(x\right)\right]\frac{H}{2}=\sum_{r=1}^{n}\tau_{i,r}\left(t\right)\vartheta_{i,r}\left(x\right)$$

where $\vartheta_{i,r}\left(x\right)=\frac{H}{2}\frac{\partial^{2}\phi_{i,r}\left(x\right)}{\partial x^{2}}$ is the strain mode shape parameter of the r-th order of beam element i.

By taking Equation (4) into Equation (5), Equation (6) can be obtained as

(6) σx=σ1σ2σ3⋮σn=∑r=1n1−ωi,r2mi+jωi,rci+Kiejωi,rtϑ1ϑ2ϑ3⋮ϑnϕ1ϕ2ϕ3⋮ϕnTF1F2F3⋮Fn

Based on Equation (6), the strain mode transfer function shown in Equation (7) can be obtained as

(7) $$H_{\sigma ,x}=\frac{\left\{\sigma_{x}\right\}}{\left\{F\right\}}=\sum_{r=1}^{n}\frac{\left\{\vartheta_{i,r}\right\}{\left\{\phi_{i,r}\right\}}^{T}}{-\omega_{i,r}^{2}m_{i}+j\omega_{i,r}c_{i}+K_{i}}e^{j\omega_{i,r}t}$$

In Equation (7), any element of the strain mode matrix of beam element i contains the modal mass $m_{i}$, modal stiffness $k_{i}$, and modal damping $c_{i}$. And it can be inferred that any row of the response of beam element i contains the information of displacement modes $\tau_{i}$, and any column contains the information of strain modes $\sigma_{i}$.

The damage index can be represented by the structural modal shape change of beam element i using Equation (8):

(8) $$\Delta \left\{\vartheta_{i,r}\right\}=\left|{\left\{\vartheta_{i,r}\right\}}_{d}-{\left\{\vartheta_{i,r}\right\}}_{u}\right|$$

where ${\left\{\vartheta_{i,r}\right\}}_{d}$ is the strain mode shape parameter of the i-th node and j-th order before damage of the beam element and ${\left\{\vartheta_{i,r}\right\}}_{u}$ is the strain mode shape parameter of the i-th node and j-th order after damage of the beam element.

According to Equations (7) and (8), the damage identification method of beam structure based on strain modal difference parameters is established:

(I) Carry out the displacement strain modal experiment of the beam structure under the double-rotor synchronous excitation condition based on the acceleration sensor and analyze and obtain the mode parameters of the beam structure $m_{i}$, $k_{i}$, $c_{i}$, and $\tau_{i}$;

(II) Measure the multi-column response modes $\sigma_{i,r}$ of element $H_{\sigma ,x}$ by the method of double-point excitation and multi-point vibration picking of the beam structure; 

(III) Solve the strain mode shape parameters $\vartheta_{i,r}$ of beam element i by numerical fitting; 

(IV) Calculate the absolute difference of strain modes between the damaged structure and the healthy structure, determine the damage position of the beam, and complete the structural damage detection of the frame beam.

## 3. Damage Identification Examples for Mine Screen Frame Beam Element

### 3.1. Physical Experimental Model of the Example

**Figure 1:**
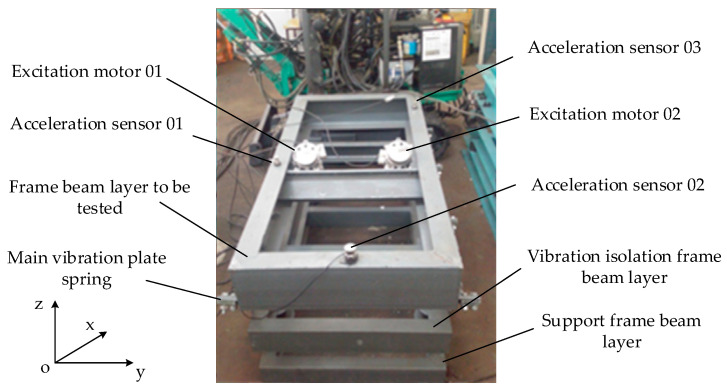
Structure and sensor arrangement of mine screen frame beam.

In order to verify the effectiveness of the strain modal difference damage identification algorithm, the double excitation mine screen frame beam shown in Figure 1 is taken as the analysis object. The three-layer frame beam in Figure 1 is made of C45E4 material, which is widely used in construction, mining, aerospace, and other industries and is universal and representative. Therefore, taking this frame beam structure as an example, using the strain modal difference mode analysis method to evaluate and monitor damage and eliminate potential safety hazards has important practical significance [23,24].

The structure of the upper layer in Figure 1 is a frame beam to be detected, which is made of 16A# (160 × 63 × 6.5 mm) channel steel. The frame beam layer to be detected (2560 × 640 × 80 mm) is connected to the vibration isolation frame beam layer (2560 × 640 × 80 mm) through the main vibration plate spring, and the vibration isolation frame beam layer is connected with the support frame beam layer fixed on platform (2560 × 640 × 50 mm) through the spiral support spring. The waist thickness of the frame beam to be tested is 9.5 mm, the leg thickness is 10 mm, the cross section is $21.96\;{mm}^{2}$, the elastic modulus is $E=2.06\times 10^{5}\;Pa$, the yield strength is $\sigma_{s}=355\;MPa$, the tensile strength is $\sigma_{b}=600\;MPa$, the Poisson ratio is υ=0.266, and the density is $\rho =7.85\times 10^{3}\;kg/m^{3}$.

In Figure 1, two excitation motors are used to excite the frame beam layer to be tested in reverse and synchronously (the frequency bandwidth of double excitation motors is 0~150 Hz), and the frame beam is a two-degree-of-freedom system in the horizontal and vertical directions. Three acceleration sensors (model: 4801A; sensitivity: 1000 mV/g; supplier: Sensor Way Measurement and Control Technology Co., Ltd., Beijing, China) are installed on the frame beam layer to detect and measure the vibration response of the frame beam.

### 3.2. Experimental Results and Analysis of Damage Identification of the Example

**Figure 2:**
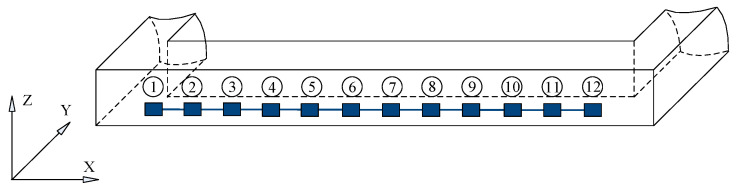
Distribution position of 12 strain gauges for mine screen frame beam strain detection.

In the experiment, the left beam of the frame beam layer to be tested in Figure 1 is divided into 12 equal parts along the x-direction, and strain gauges are attached according to the positions shown in Figure 2 (model: BX120-10AA; resistance: 120 Ω; sensitivity coefficient: 2.08; sensitive grid size: 10 × 2 mm; base size: 14.5 × 4.5 mm; supplier: Yiyang Vibration Testing Technology Co., Ltd., Beijing, China). Strain modal response experiments had been carried out to analyze the strain modal parameters of the frame beam. The sampling frequency is set at 2 kHz, with 8192 sampling points in the time domain and 4096 sampling points in the frequency domain.

According to the verification of the damage detection algorithm of the frame beam, prefabricated damage defects were adopted. A transverse groove with a width of 0.01 mm was cut at the position of No. 3 strain gauge of the frame beam by a cutting machine to simulate crack damage, and the crack depth was cut into three damage conditions: 1.0 mm (10% of the thickness of frame beam to be tested), 3.0 mm (30% of the thickness of frame beam to be tested), and 5.0 mm (50% of the thickness of frame beam to be tested). According to three working conditions, the damage identification experiment of the frame beam is carried out by using the strain modal difference algorithm. Table 1 is the experimental measurement data of the modal frequency of the ore screen frame beam after pre-damage.

As can be seen from Table 1, compared with the modal frequency value of the frame beam under the nondestructive condition, the modal frequency of the same order has little change under the pre-damage condition, and the change amount is less than 10%, which indicates that the frequency change in the system is not sensitive to the damage of the frame beam structure. From Table 1 as a whole, the modal frequency shows a decreasing trend with the increase in the damage amount. Although the decreasing trend is not obvious, the damage to the frame beam structure can be preliminarily identified by the change in modal frequency, and the damage degree can be qualitatively identified. Because the index change value is small, it cannot accurately locate the damage position, especially when multiple damages occur at the same time. It is even more impossible to accurately locate the modal frequency change, which shows that the modal frequency of the frame beam structure reflects the overall response characteristics of the frame beam, cannot reflect the local characteristics of the frame beam structure, and is insensitive to the identification of minor damages. Therefore, the damage identification experiment of strain modal difference was carried out on this basis in this paper.

**Figure 3:**
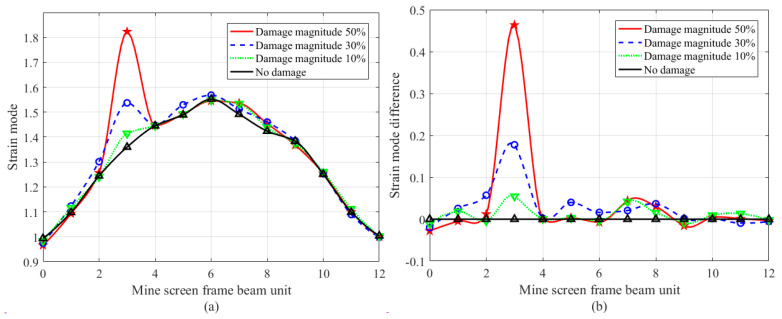

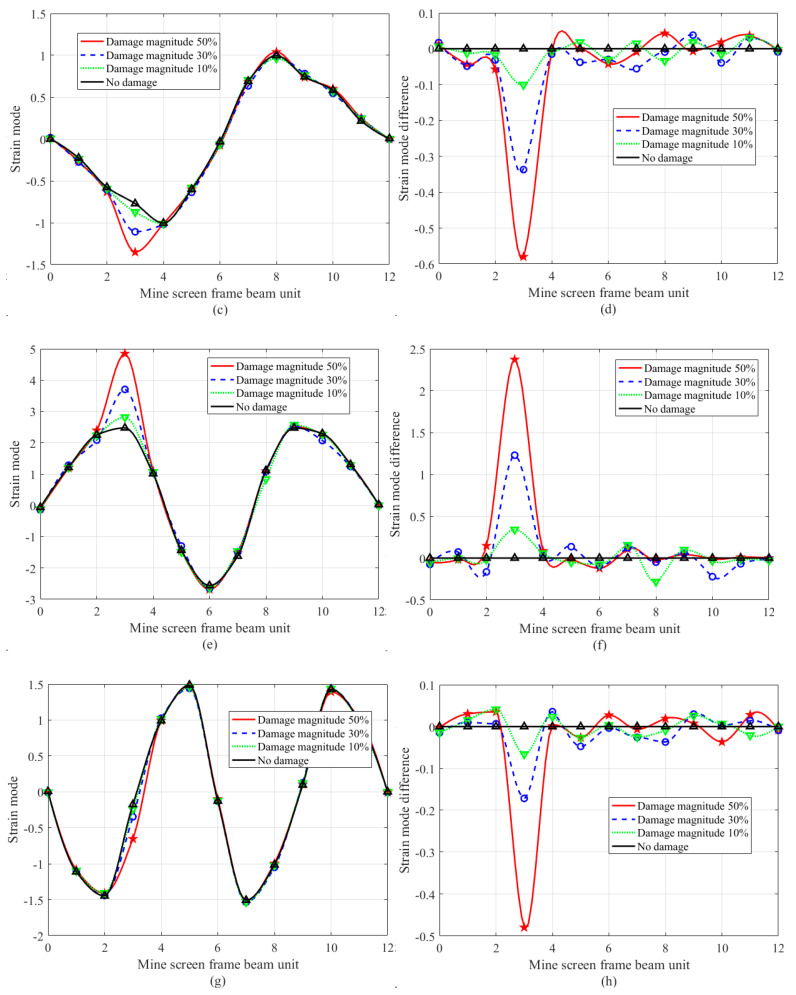
First four order strain modes and strain mode difference curves of a frame beam with single damage position, in which circles/stars/triangles represent the measured point data under four working conditions: (**a**) 1st order strain mode; (**b**) 1st order strain mode difference; (**c**) 2nd order strain mode; (**d**) 2nd order strain mode difference; (**e**) 3rd order strain mode; (**f**) 3rd order strain mode difference; (**g**) 4th order strain mode; (**h**) 4th order strain mode difference.

In order to quantitatively judge the damage location and damage degree of the frame beam in Figure 1, based on the strain modal difference algorithm of Equations (7) and (8), the first four modes and modal differences of the damage element were obtained as shown in Figure 3.

It can be seen from Figure 3 that in the first four-order strain modal curves of a single damage location, except for a small deformation change at the damage element (compared with the frame beam structure size of Figure 1, the change amount is 6.89 mm, and the change rate is 0.269%), the strain modal curves at other elements are relatively smooth. Strain modal difference curves show obvious abrupt change at the damage element, and the abrupt change value increases with the increase in the damage degree. Taking the fourth-order modal curve shown in Figure 3g as an example, there is no obvious change in the damage element curve because the damage position is at the inflection point of the curve, but the strain modal difference curve shown in Figure 3h has obvious abrupt change at this position, and the abrupt change position is consistent with the experimental setting position. It shows that the research method in this paper is effective for single damage identification of frame beam elements.

**Figure 4:**
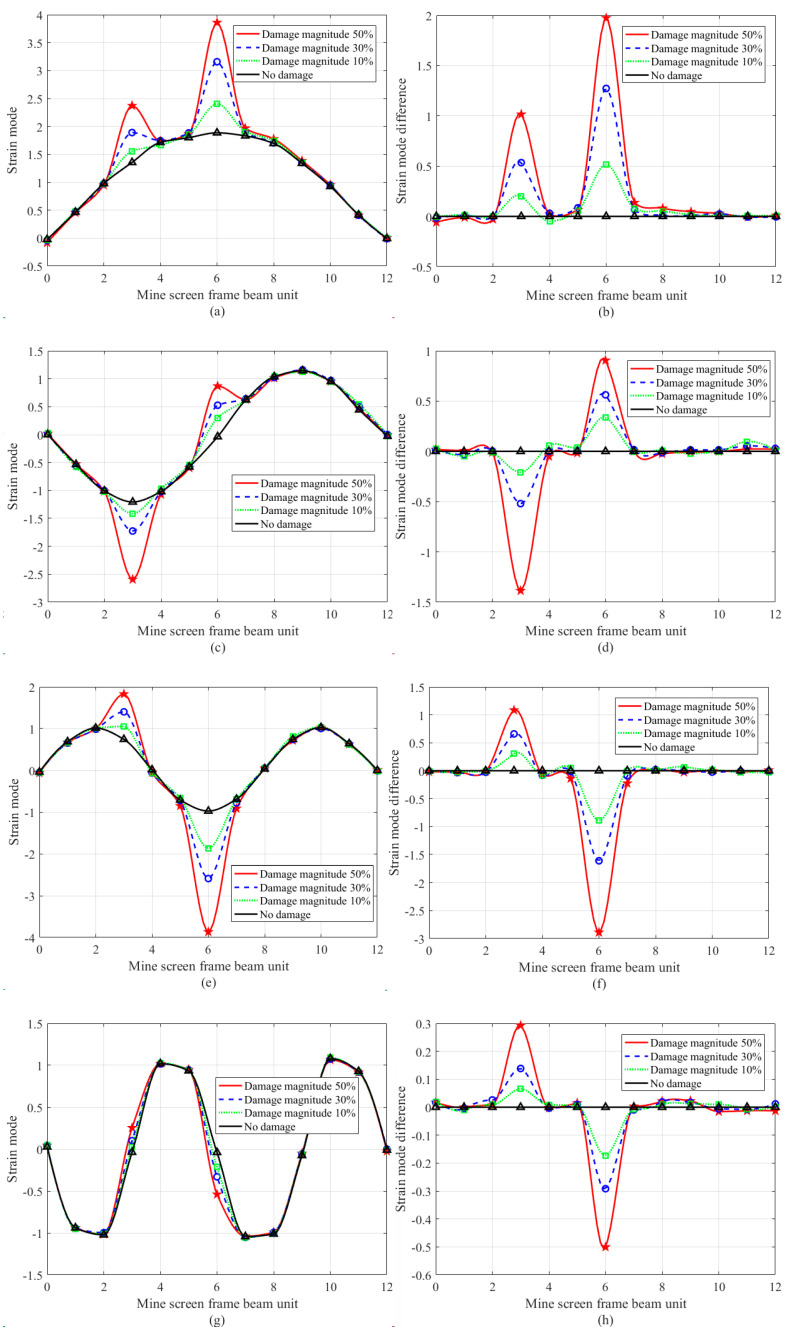
First four order strain modes and strain mode difference curves of a frame beam with double damage positions, in which circles/stars/triangles represent the measured point data under four working conditions: (**a**) 1st order strain mode; (**b**) 1st order strain mode difference; (**c**) 2nd order strain mode; (**d**) 2nd order strain mode difference; (**e**) 3rd order strain mode; (**f**) 3rd order strain mode difference; (**g**) 4th order strain mode; (**h**) 4th order strain mode difference.

In order to further verify the effectiveness of the proposed method in engineering applications, double damage identification experiments were carried out in this paper. Similar to the single damage test, pre-damage defects are taken at the position of the No. 3 strain gauge and the No. 6 strain gauge of the frame beam in Figure 2. The transverse grooves with a width of 0.01 mm were cut at the No. 3 strain gauge patch position and the No. 6 strain gauge patch position of the frame beam in Figure 1 to simulate crack damage, and the crack depth was cut into three damage conditions: 1.0 mm (10% of the thickness of the frame beam to be detected), 3.0 mm (30% of the thickness of the frame beam to be detected), and 5.0 mm (50% of the thickness of the frame beam to be detected). According to three working conditions, using the strain modal difference algorithm, the damage identification results of the frame beam to be detected in Figure 4 are obtained.

It can be seen from Figure 4 that in the first four-order strain modal difference curves, both node 3 and node 6 have obvious abrupt changes, while other nodes are relatively gentle, so it can be clearly judged that the damage elements are node 3 and node 6, and like the single damage experiment in Figure 3 and Figure 4, can accurately reflect the damage degree of the two nodes.

In order to discuss the strain modal variation in the frame beam under damage condition, based on Figure 3 and Figure 4, the statistical results of the strain modal difference at the damage position are given in Table 2.

Table 2 illustrates that in the dual damage identification of node 3 and node 6, in the first-order mode, the mutation values for 10% damage are 0.2023 and 0.5194, respectively; for 30% damage, the values are 0.5342 and 1.2729, respectively; and for 50% damage, the values are 1.0156 and 1.9741, respectively. As the extent of the damage escalates, the mutation values of the strain mode differences at the damage location likewise correspondingly grow. The deformation of the frame beam at node 6 exceeds that at node 3, resulting in a greater amplitude of the strain mode at node 6 compared to node 3. Likewise, a comparable rule of change exists in the second-order, third-order, and fourth-order modes, enabling precise identification of structural damage in this context. The strain mode difference vibration pattern analysis method demonstrates greater sensitivity in identifying damage in a single material compared to traditional methods. It effectively assesses the damaged condition of the mine screen frame beam structure. The damage identification technique based on strain mode difference presented in this paper is applicable for practical engineering applications concerning the damage assessment of steel structures.

## 4. Conclusions

This paper analyzed the displacement mode, strain mode, and parameter identification methodology of beam structures by investigating the damage identification technology of mine screen beams subjected to synchronous excitation from dual rotors, effectively identifying the vibration mode, frequency, damping, and other modal parameters of the mine screen’s frame beam under synchronous excitation conditions. The method can be applied to identify strain modal characteristics of analogous frame beam constructions in engineering.

The research presented in this paper indicates that, when subjected to vibrational excitation, the intrinsic modal frequency of the ore screen frame beam structure exhibits insensitivity to structural damage. However, the strain mode difference shape experiences a pronounced abrupt change at the damage site, enabling precise identification of the damage location. Furthermore, under identical damage conditions of the beam structure, the higher-order strain mode difference mode shape demonstrates greater sensitivity to the extent of damage, with the magnitude of the difference mutation increasing in correlation with the severity of the damage.

The example application in this paper demonstrates a certain linear correlation between the damage degree of the steel frame beam structure and the abrupt variation in its strain modal difference. In engineering practice, according to the pre-established frame beam structural damage evaluation criteria, the structural damage location identification of the mine screen frame beam can be carried out and the degree of damage can be determined to assess the health status of the beam structure. The study methodology proposed in this paper possesses significant engineering applicable value for the identification of structural damage and the preservation of health in mine screen frame beams.

## Figures and Tables

**Table 1:** Experimental data of mine screen frame beam structure under single pre-damage condition.

Frequency Order	No Damage	Working Condition 1		Working Condition 2		Working Condition 3	
	Modal Frequency	Modal Frequency	Change Ratio	Modal Frequency	Change Ratio	Modal Frequency	Change Ratio
1	9.86	9.73	1.32%	9.51	3.55%	9.16	7.10%
2	12.30	12.12	1.46%	11.83	3.82%	11.16	9.27%
3	18.58	18.33	1.35%	17.96	3.34%	17.18	7.53%
4	25.49	24.86	2.47%	24.13	5.34%	23.27	8.71%
5	29.91	29.07	2.81%	28.32	5.32%	27.34	8.59%
6	37.69	37.11	1.54%	36.02	4.43%	34.25	9.13%

**Table 2:** Experimental data of mine screen frame beam structure under double pre-damage conditions.

Modal	Working Condition 1		Working Condition 2		Working Condition 3	
	Mutation Value	Mutation Ratio/%	Mutation Value	Mutation Ratio/%	Mutation Value	Mutation Ratio/%
1st	0.2023	14.93	0.5342	39.41	1.0156	74.93
	0.5194	27.55	1.2729	67.52	1.9741	104.72
2nd	−0.2096	17.37	−0.5196	43.06	−1.3862	1148.75
	0.3378	931.03	0.5625	1550.46	0.9049	2494.48
3rd	0.3082	41.49	0.6593	88.74	1.0856	146.12
	−0.8928	91.43	−1.6119	165.08	−2.8898	295.96
4th	0.0656	174.48	0.1385	368.46	0.2925	778.41
	−0.1735	441.39	−0.2917	741.99	−0.5006	1273.25

## Data Availability

The original contributions presented in this study are included in this article.
